# Measuring Connectivity in Linear Multivariate Processes: Definitions, Interpretation, and Practical Analysis

**DOI:** 10.1155/2012/140513

**Published:** 2012-05-14

**Authors:** Luca Faes, Silvia Erla, Giandomenico Nollo

**Affiliations:** Laboratorio Biosegnali, Dipartimento di Fisica & BIOtech, Università di Trento, via delle Regole 101, 38123 Mattarello, Trento, Italy

## Abstract

This tutorial paper introduces a common framework for the evaluation of widely used frequency-domain measures of coupling (coherence, partial coherence) and causality (directed coherence, partial directed coherence) from the parametric representation of linear multivariate (MV) processes. After providing a comprehensive time-domain definition of the various forms of connectivity observed in MV processes, we particularize them to MV autoregressive (MVAR) processes and derive the corresponding frequency-domain measures. Then, we discuss the theoretical interpretation of these MVAR-based connectivity measures, showing that each of them reflects a specific time-domain connectivity definition and how this results in the description of peculiar aspects of the information transfer in MV processes. Furthermore, issues related to the practical utilization of these measures on real-time series are pointed out, including MVAR model estimation and significance assessment. Finally, limitations and pitfalls arising from model mis-specification are discussed, indicating possible solutions and providing practical recommendations for a safe computation of the connectivity measures. An example of estimation of the presented measures from multiple EEG signals recorded during a combined visuomotor task is also reported, showing how evaluation of coupling and causality in the frequency domain may help describing specific neurophysiological mechanisms.

## 1. Introduction

Multivariate (MV) time series analysis is nowadays extensively used to investigate the concept of connectivity in dynamical systems. Connectivity is evaluated from the joint description of multiple time series collected simultaneously from the considered system. Applications of this approach are ubiquitous in the analysis of experimental time series recorded in various research fields, ranging from economics to biomedical sciences. In neuroscience, the concept of brain connectivity [1] plays a central role both in the understanding of the neurophysiological mechanisms of interaction among different areas of the brain, and in the development of indexes for the assessment of mechanism impairment in pathological conditions (see, e.g., [2] and references therein). The general term “brain connectivity” encompasses different modes, each making reference to specific aspects of how brain areas interact. In particular, “functional connectivity” refers to evaluation of statistical dependencies between spatially distributed neuronal units, while “effective connectivity” refers to the description of networks of directional effects of one neural unit over another [3]. In the context of time series analysis, the notions of functional and effective connectivity can be investigated, respectively, in terms of *coupling*, that is, the presence of interactions, and of *causality*, that is, the presence of driver-response relationships, between two neurophysiological time series taken from the available MV data set.

The assessment of coupling and causality in MV processes may be performed following either linear or nonlinear time series analysis approaches [2, 4]. While nonlinear methods are continuously under development [5–10] and offer the intriguing possibility of studying complex signal interactions, linear signal processing approaches [11] are extensively used in MV neurophysiological time series analysis. The main reason for the popularity of linear methods lies in the fact that they are strictly related to the frequency-domain representation of multichannel data [12, 13], and thus, lend themselves to the representation of biological signals which are rich of oscillatory content. In physiological systems, the linear frequency-domain representation favors the characterization of connectivity between specific oscillatory components such as the EEG rhythms [14].

In the linear signal processing framework, connectivity is very often formalized in the context of an MV autoregressive (MVAR) representation of the available time series, which allows to derive time- and frequency-domain pictures, respectively, by the model coefficients and by their spectral representation. Accordingly, several frequency-domain measures of connectivity have been introduced and applied in recent years. Coupling is traditionally investigated by means of the coherence (Coh) and the partial coherence (PCoh), classically known, for example, from Kay [15] or Bendat and Piersol [16]. Measures able to quantify causality in the frequency-domain were proposed more recently, the most used being the directed transfer function (DTF) [12, 17], the directed coherence (DC) [18], and the partial directed coherence (PDC) [19], the latter repeatedly refined after its original formulation [20–22]. These measures are widely used for the analysis of interactions among physiological time series, and—in particular—to characterize brain connectivity [23–31]. Recent studies have proposed deeper interpretation of frequency-domain connectivity measures [21, 32], as well as comparison on both simulated and real physiological time series [11, 33]. Despite this large body of work, the interpretation of frequency-domain coupling and causality measures is not always straightforward, and this may lead to erroneous descriptions of connectivity and related mechanisms. Examples of ambiguities emerged in the interpretation of these measures are the debates about the ability of PCoh to measure some forms of causality [34, 35], about the specific kind of causality which is reflected by the DTF and DC measures [17, 19, 36], and about whether the PDC could be suitably re-normalized to make its modulus able to reflect meaningfully the strength of coupling [22, 32].

In order to settle these interpretability issues, a joint description of the different connectivity measures, as well as a contextualization in relation to well-defined time-domain concepts, is required. According to this need, the present paper has a tutorial character such that—instead of proposing new measures—it is aimed to enhance the interpretability and favour the utilization of existing frequency-domain connectivity measures based on MVAR modelling. To this end, we introduce a common framework for the evaluation of Coh, PCoh, DC/DTF, and PDC from the frequency-domain representation of MVAR processes, which is exploited to relate the various measures to each other as well as to the specific coupling or causality definition which they underlie. After providing a comprehensive definition of the various forms of connectivity observed in MV processes, we particularize them to MVAR processes and derive the corresponding frequency-domain measures. Then, we discuss the theoretical interpretation of these measures, showing how they are able to describe peculiar aspects of the information transfer in MV time series. Further, we point out issues related to practical estimation, limitations, and recommendations for the utilization of these measures on real MV time series. An example of estimation of the presented measures from multiple EEG signals recorded during a sensorimotor integration experiment is finally presented to illustrate their practical applicability.

## 2. Connectivity Definitions in the Time Domain

### 2.1. Multivariate Closed-Loop Processes

Let us consider *M* stationary stochastic processes *y*_*m*_, *m* = 1,…, *M*, collected in the multivariate (MV) vector process **Y** = [*y*_1_,…,*y*_*M*_]^T^. Without loss of generality, we assume that the processes are real-valued, defined at discrete time (*y*_*m*_ = {*y*_*m*_(*n*)}; e.g., are sampled versions of the continuous time processes *y*_*m*_(*t*), taken at the times *t*_*n*_ = *nT*, with *T* the sampling period) and have zero mean (E[*y*_*m*_(*n*)] = 0, where E[·] is the statistical expectation operator). An MV closed loop vector process of order *p* is defined expressing the present value of each scalar process, *y*_*m*_(*n*), as a function of the *p* past values of all processes, collected in *Y*_*l*_ = {*y*_*l*_(*n* − 1),…, *y*_*l*_(*n* − *p*)}  (*l*, *m* = 1,…, *M*):

(1) $$y_{m}\left(n\right)=f_{m}\left(Y_{1},\ldots ,Y_{M}\right)+u_{m}\left(n\right),$$

where *u*_*m*_ are independent white noise processes describing the error in the representation. Note that the definition in (1) limits to past values only the possible influences of one process to another, excluding instantaneous effects (i.e., effects occurring within the same lag). The absence of instantaneous effects is denoted as strict causality of the closed loop MV process [37, 38] and will be assumed henceforth. 

Given two processes *y*_*i*_ and *y*_*j*_ of the closed-loop, the general concept of *connectivity* can be particularized to the study of *causality* or *coupling* between *y*_*i*_ and *y*_*j*_, which investigate, respectively, directional or non-directional properties of the considered pairwise interaction. With the aim of supporting interpretation of the frequency-domain connectivity measures presented in Section 3, we state now specific time-domain definitions of coupling and causality valid for an MV closed-loop process (see Table 1). *Direct causality* from *y*_*j*_ to *y*_*i*_, *y*_*j*_ → *y*_*i*_, exists if the prediction of *y*_*i*_(*n*) based on {*Y*_1_,…, *Y*_*M*_} is better (i.e., yields a lower prediction error) than the prediction of *y*_*i*_(*n*) based on {*Y*_1_,…, *Y*_*M*_}∖*Y*_*j*_. *Causality* from *y*_*j*_ to *y*_*i*_, *y*_*j*_⇒*y*_*i*_, exists if a cascade of direct causality relations *y*_*j*_ → *y*_*m*_ ⋯ →*y*_*i*_ occurs for at least one *m* ∈ {1,…, *M*}; if *m* = *i* or *m* = *j* causality reduces to direct causality, while for *m* ≠ *i*, *m* ≠ *j*, the causality relation is indirect. *Direct coupling* between *y*_*i*_ and *y*_*j*_, *y*_*i*_↔*y*_*j*_, exists if *y*_*i*_ → *y*_*j*_ or *y*_*j*_ → *y*_*i*_. *Coupling* between *y*_*i*_ and *y*_*j*_, *y*_*i*_⇔*y*_*j*_, exists if *y*_*i*_⇒*y*_*j*_ or *y*_*j*_⇒*y*_*i*_. The rationale of these connectivity definitions is grounded on the very popular notion of Granger causality, as originally introduced by the seminal paper of Granger for a bivariate closed loop linear stochastic process [39], and on intuitive generalizations aimed at moving from the study of causality to the study of coupling, and from bivariate (*M* = 2) to MV (*M* ≥ 3) processes. Specifically, our definition of direct causality agrees with the Granger's original statement [39] for bivariate processes, and with the notion of *prima facie* Granger causality introduced later in [40] for multivariate processes. The definition of causality is a generalization incorporating both direct and indirect causal influences from one process to another, while the coupling definitions generalize the causality definitions by accounting for both forward and backward interactions.

In addition to the definitions provided above, we state the following definitions of coupling, which are referred to as spurious because they concern a mathematical formalism rather than an intuitive property of two interacting processes: *spurious direct coupling* between *y*_*i*_ and *y*_*j*_ exists if *y*_*i*_ → *y*_*m*_ and *y*_*j*_ → *y*_*m*_ for at least one *m* ∈ {1,…, *M*}, *m* ≠ *i*, *m* ≠ *j*; *spurious coupling* between *y*_*i*_ and *y*_*j*_ exists if *y*_*m*_⇒*y*_*i*_ and *y*_*m*_⇒*y*_*j*_ for at least one *m* ∈ {1,…, *M*}, *m* ≠ *i*, *m* ≠ *j*. These definitions suggest that two processes can be interpreted as directly coupled also when they both directly cause a third common process, and as coupled also when they are both caused by a third common process, respectively, and are introduced here to provide a formalism for explaining a confounding property of the two common frequency-domain coupling measures reviewed in Section 3.

**Figure 1:**
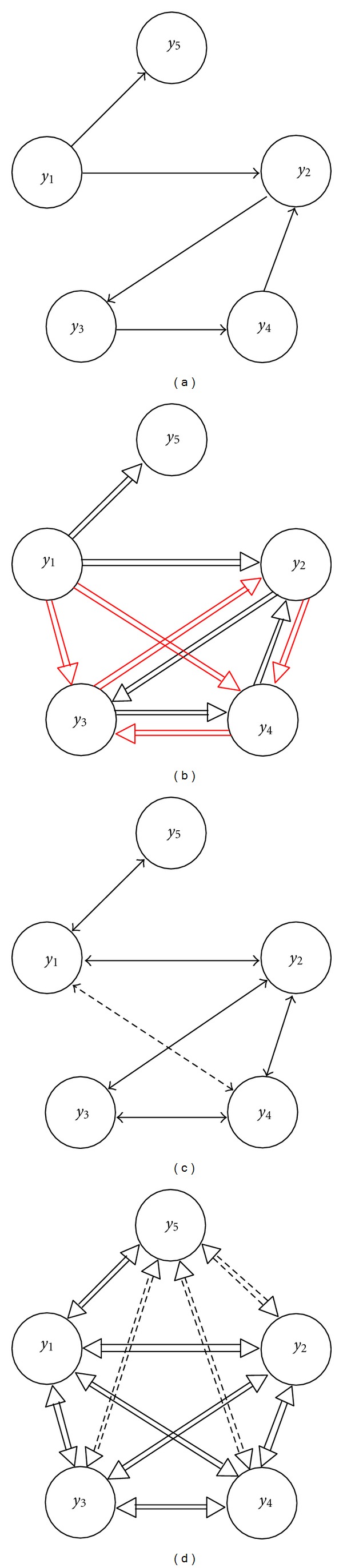
Graphical models for an illustrative five-dimensional closed loop process, denoting the scalar processes (*y*_*i*_, *i* = 1,…, 5) as graph nodes and the connectivity relations between processes as connecting arrows. Graphs depict an imposed set of direct causality relations (*y*_*j*_ → *y*_*i*_, (a)), as well as the corresponding sets of causality (*y*_*j*_⇒*y*_*i*_, (b)), direct coupling (*y*_*i*_↔*y*_*j*_, (c)), and coupling (*y*_*i*_⇔*y*_*j*_, (d)) relations. Indirect causality relations are depicted with red arrows in (b), while spurious direct coupling and spurious coupling relations are depicted with dashed double-head arrows in ((c) and (d)).

An illustrative example of the described causality and coupling relations is reported in Figure 1, showing a network of *M* = 5 interacting processes where each node represents a process and the connecting arrows represent coupling or causality relations. The structure of the process is unambiguously determined by the direct causality relations set in Figure 1(a), that is, *y*_1_ → *y*_2_, *y*_2_ → *y*_3_, *y*_3_ → *y*_4_, *y*_4_ → *y*_2_, and *y*_1_ → *y*_5_. All other connectivity definitions can be established from this set of direct causality effects. Indeed, the causality relations follow from the presence of either direct causality (Figure 1(b), black arrows) or indirect causality (Figure 1(b), red arrows). Direct coupling exists as a consequence of direct causality (Figure 1(c), solid arrows), and also as a consequence of the common driving exerted by *y*_1_ and *y*_4_ on *y*_2_, such that the spurious connection *y*_1_↔*y*_4_ (Figure 1(c), dashed arrow) arises. Finally, coupling is detected between each pair of processes: while most relations derive from the causality effects (Figure 1(d), solid arrows), the relations *y*_2_⇔*y*_5_, *y*_3_⇔*y*_5_, and *y*_4_⇔*y*_5_ are spurious as they derive from the common driving exerted by *y*_1_ on *y*_2_ and *y*_5_, on *y*_3_ and *y*_5_, and on *y*_4_ and *y*_5_ (Figure 1(d), dashed arrows).

### 2.2. Multivariate Autoregressive Processes

In the linear signal processing framework, the MV closed-loop process **Y**(*n*) = [*y*_1_(*n*),…,*y*_*M*_  (*n*)]^T^ can be represented as the output of a MV linear shift-invariant filter [15]:

(2) $$Y\left(n\right)=\overset{\infty }{\underset{k=-\infty }{\sum }}H\left(k\right)U\left(n-k\right),$$

where **U**(*n*) = [*u*_1_(*n*)⋯*u*_*M*_(*n*)]^T^ is a vector of *M* zero-mean input processes and **H**(*k*) is the *M* × *M* filter impulse response matrix. A very common representation of (2), extensively used in time series analysis, is the MV autoregressive (MVAR) representation [15]:

(3) $$Y\left(n\right)=\overset{p}{\underset{k=1}{\sum }}A\left(k\right)Y\left(n-k\right)+U\left(n\right),$$

where **A**(*k*) are *M* × *M* coefficient matrices in which the element *a*_*ij*_(*k*) describes the dependence of *y*_*i*_(*n*) on *y*_*j*_(*n* − *k*) (*i*, *j* = 1,…*M*; *k* = 1,…, *p*). Note that (3) is a particularization of (1) in which each function *f*_*m*_ is a linear first-order polynomial. The input process **U**(*n*), also called innovation process, is assumed to be composed of white and uncorrelated noises; this means that the correlation matrix of **U**(*n*), **R**_**U**_(*k*) = E[**U**(*n*)**U** ^T^(*n* − *k*)], is zero for each lag *k* > 0, while it is equal to the covariance matrix Σ = cov⁡(**U**(*n*)) for *k* = 0. Under the assumption of strict causality, the input white noises are uncorrelated even at lag zero, so that their covariance reduces to the diagonal matrix Σ = diag⁡(*σ* ^2^_*i*_).

One major benefit of the representation in (3) is that it allows to investigate properties of the joint description of the processes *y*_*m*_ from the model coefficients. In fact, the connectivity definitions provided in Section 2.1 for a general closed-loop MV process can be specified for an MVAR process in terms of the elements of **A**(*k*). Conceptually, causality and coupling relations are found when the pathway relevant to the interaction is active, that is, is described by nonzero coefficients in **A** (see Table 1). More formally, we have that *y*_*j*_ → *y*_*i*_ if *a*_*ij*_(*k*) ≠ 0 for at least one *k* ∈ {1,…, *p*}; *y*_*j*_⇒*y*_*i*_ if *a*_*m*_*l*_*m*_*l*−1__(*k*_*l*_) ≠ 0 for at least one set of *L* + 1 different values for *m*_*l*_ ∈ {1,…, *M*} with *m*_0_ = *j*, *m*_*L*_ = *i*, and one set of *L* lags *k*_*l*_ ∈ {1,…, *p*}(*l* = 1,…, *L*; 1 ≤ *L* < *M*); *y*_*i*_↔*y*_*j*_ if, for at least and one pair *k*_1_, *k*_2_ ∈ {1,…, *p*}, one of the following holds: (i) *a*_*ji*_(*k*_1_) ≠ 0 or *a*_*ij*_(*k*_2_) ≠ 0 (direct coupling), or (ii) *a*_*mi*_(*k*_1_) ≠ 0 and *a*_*mj*_(*k*_2_) ≠ 0 for at least one *m* ∈ {1,…, *M*} such that *m* ≠ *i*, *m* ≠ *j* (spurious direct coupling); *y*_*i*_⇔*y*_*j*_ if, for some *m*_*l*_ ∈ {1,…, *M*} and *k*_*l*_ ∈ {1,…, *p*} one of the following holds: (i) *a*_*m*_*l*_*m*_*l*−1__(*k*_*l*_) ≠ 0 with either *m*_0_ = *i*, *m*_*L*_ = *j* or *m*_0_ = *j*, *m*_*L*_ = *i* (coupling), or (ii) *a*_*m*_*l*_*m*_*l*−1__(*k*_*l*_) ≠ 0 with both *m*_0_ = *m*, *m*_*L*_ = *i* and *m*_0_ = *m*, *m*_*L*_ = *j* for at least one *m* ∈ {1,…, *M*} such that *m* ≠ *i*, *m* ≠ *j* (spurious coupling).

**Figure 2:**
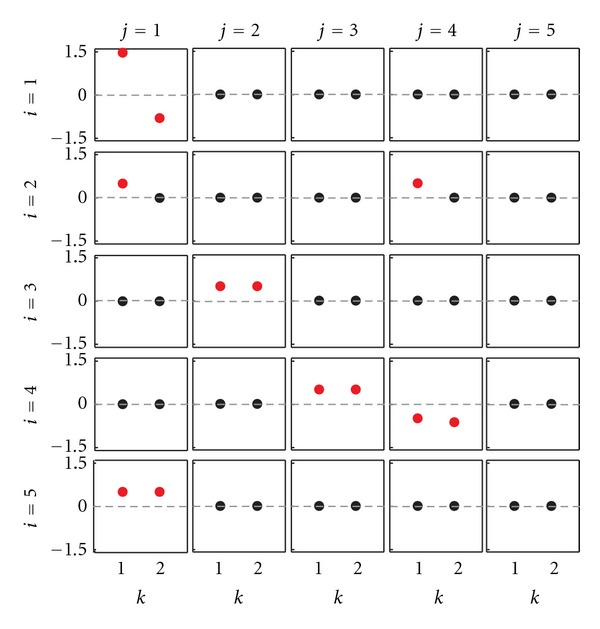
Time-domain connectivity pattern for the illustrative MVAR process of (4). Each plot depicts the values set for the coefficients *a*_*ij*_(*k*) (*i*, *j* = 1,…, 5; *k* = 1,2), with nonzero coefficients evidenced in red.

To illustrate these time-domain connectivity definitions, let us consider the MVAR process of dimension *M* = 5 and order *p* = 2:

(4) $$\begin{array}{cc} y_{1}\left(n\right) & =2\rho_{1}\cos\left(2\pi f_{1}\right)y_{1}\left(n-1\right)-\rho_{1}^{2}y_{1}\left(n-2\right)+u_{1}\left(n\right),\\ y_{2}\left(n\right) & =0.5y_{1}\left(n-1\right)+0.5y_{4}\left(n-1\right)+u_{2}\left(n\right),\\ y_{3}\left(n\right) & =0.5y_{2}\left(n-1\right)+0.5y_{2}\left(n-2\right)+u_{3}\left(n\right),\\ y_{4}\left(n\right) & =2\rho_{4}\cos\left(2\pi f_{4}\right)y_{4}\left(n-1\right)-\rho_{4}^{2}y_{4}\left(n-2\right)\\ & \;+0.5y_{3}\left(n-1\right)+0.5y_{3}\left(n-2\right)+u_{4}\left(n\right),\\ y_{5}\left(n\right) & =0.5y_{1}\left(n-1\right)+0.5y_{1}\left(n-2\right)+u_{5}\left(n\right), \end{array}$$

with *ρ*_1_ = 0.9, *f*_1_ = 0.1, *ρ*_4_ = 0.8, *f*_4_ = 0.3, where the inputs *u*_*i*_(*n*) are fully uncorrelated and with variance *σ* ^2^_*i*_ = 1(*i* = 1,…, 5). Equation (4) defines one of the possible MVAR processes realizing the connectivity patterns depicted in Figure 1. The matrix layout plot of Figure 2, depicting the values set for the coefficients *a*_*ij*_(*k*), provides a straightforward interpretation of connectivity in the time domain. In fact, non-zero values in the coefficient matrices **A**(1) and **A**(2) determine direct causality and causality among the processes—and consequently direct coupling and coupling—in agreement with the definitions provided above. In particular, we note that direct causality from *y*_*j*_ to *y*_*i*_ occurs if at least one coefficient in the (*i*, *j*)th plot of the matrix layout of Figure 2 is nonzero (red symbols). For example, nonzero values of *a*_21_(1) and of {*a*_32_(1), *a*_32_(2)} determine the direct causality relations *y*_1_ → *y*_2_ and *y*_2_ → *y*_3_ when considered separately, as well as the causality relation *y*_2_⇒*y*_3_ (indirect effect) when considered together; nonzero values of *a*_21_(1) and of *a*_24_(1) determine the direct coupling relations *y*_1_↔*y*_2_ and *y*_2_↔*y*_4_, and also the spurious direct coupling *y*_1_↔*y*_4_; nonzero values of {*a*_21_(1), *a*_32_(1), *a*_32_(2)} and of {*a*_51_(1), *a*_51_(2)} determine the coupling relations *y*_1_⇔*y*_3_ and *y*_1_⇔*y*_5_, but also the spurious coupling *y*_3_⇔*y*_5_. Note that the diagonal values of **A**(*k*) do not provide direct information on connectivity, but rather determine autonomous oscillations in the processes. In this case, narrow-band oscillations are generated for the process *y*_*i*_ by setting complex-conjugate poles with modulus *ρ*_*i*_ and phases ±2*πf*_*i*_ (i.e., imposing *a*_*ii*_(1) = 2*ρ*_*i*_cos⁡⁡(2*πf*_*i*_) and *a*_*ii*_(2) = −*ρ*_*i*_^2^, *i* = 1,4).

## 3. Connectivity Definitions in the Frequency Domain

### 3.1. Connectivity Measures

The derivation of connectivity measures which reflect and quantify in the frequency domain the time-domain definitions provided in Section 2 proceeds in two steps: first, the known correlation and partial correlation time-domain analyses are transposed in the frequency domain to describe the concepts of coupling and direct coupling, respectively; second, the parametric representation of the process is exploited to decompose the derived spectral measures of (direct) coupling into measures of (direct) causality. As to the first step, time-domain interactions within the MV closed-loop process **Y**(*n*) may be characterized by means of the time-lagged correlation matrix **R**(*k*) = *E*[**Y**(*n*)**Y** ^T^(*n* − *k*)] and of its inverse **R**(*k*)^−1^, whose elements may be used to define the so called correlation coefficient and partial correlation coefficient between two processes *y*_*i*_ and *y*_*j*_ [41]:

(5) $$\begin{array}{c} \rho_{ij}\left(k\right)=\frac{r_{ij}\left(k\right)}{\sqrt{r_{ii}\left(k\right)r_{jj}\left(k\right)}},\\ \begin{array}{c} \eta_{ij}\left(k\right)=-\frac{p_{ij}\left(k\right)}{\sqrt{p_{ii}\left(k\right)p_{jj}\left(k\right)}}, \end{array} \end{array}$$

where *r*_*ij*_(*k*) and *p*_*ij*_(*k*) are the *i*-*j* elements of **R**(*k*) and **R**(*k*)^−1^. The correlation *ρ*_*ij*_ is a normalized measure of the linear interdependence between *y*_*i*_(*n*) and *y*_*j*_(*n* − *k*) and, as such, quantifies coupling in the time-domain. The partial correlation *η*_*ij*_ is a measure of direct coupling, in the sense that it quantifies the linear interdependence between *y*_*i*_(*n*) and *y*_*j*_(*n* − *k*) after removing the effects of all remaining processes, according to a procedure denoted as partialization [42]. The frequency-domain counterpart of these measures is obtained considering the traditional spectral analysis of MV processes on one side [15], and the corresponding dual analysis performed in the inverse spectral domain on the other side [43]. Specifically, the *M* × *M* spectral density matrix **S**(*f*) is defined as the Fourier Transform (FT) of **R**(*k*), while the inverse spectral matrix **P**(*f*) = **S**(*f*)^−1^ results as the FT of the partial correlation matrix **R**(*k*)^−1^. The elements of the spectral matrices **S**(*f*) and **P**(*f*) are combined to define the so-called *coherence* (Coh) and *partial coherence* (PCoh) functions [44]:

(6a) $$\begin{array}{c} \Gamma_{ij}\left(f\right)=\frac{S_{ij}\left(f\right)}{\sqrt{S_{ii}\left(f\right)S_{jj}\left(f\right)}}, \end{array}$$

(6b) $$\begin{array}{c} \Pi_{ij}\left(f\right)=-\frac{P_{ij}\left(f\right)}{\sqrt{P_{ii}\left(f\right)P_{jj}\left(f\right)}}. \end{array}$$

When a closed-loop MV process is particularized to a MVAR process, the spectral representation may be obtained taking the FT of the representations in (2) and (3), which yields **Y**(*f*) = **H**(*f*)**U**(*f*) and **Y**(*f*) = **A**(*f*)**Y**(*f*) + **U**(*f*), respectively, where **Y**(*f*) and **U**(*f*) are the FTs of **Y**(*n*) and **U**(*n*), and the *M* × *M* transfer matrix and coefficient matrix are defined in the frequency domain as:

(7) $$H\left(f\right)=\overset{\infty }{\underset{k=-\infty }{\sum }}H\left(k\right)e^{-j2\pi fkT},\;\;\;A\left(f\right)=\overset{p}{\underset{k=1}{\sum }}A\left(k\right)e^{-j2\pi fkT}.$$

Comparing the two spectral representations, it is easy to show that the coefficient and transfer matrices are linked by: $H(f)={\left[I-A\left(f\right)\right]}^{-1}=\overset{̅}{A}{\left(f\right)}^{-1}$. This important relation is useful to draw the connection between the spectral density matrix **S**(*f*) and its inverse **P**(*f*), as well as to decompose the symmetric frequency-domain connectivity measures into terms eliciting directionality. The key element is the spectral factorization theorem [45]:

(8) $$S\left(f\right)=H\left(f\right)\Sigma H^{H}\left(f\right),\;\;P\left(f\right)={\overset{̅}{A}}^{\text{H}}\left(f\right)\Sigma^{-1}\overset{̅}{A}\left(f\right),$$

(where *H *stands for Hermitian transpose), which allows to represent, under the assumption of strict causality, the elements of the spectral density matrices as:

(9) $$\begin{array}{c} S_{ij}\left(f\right)=\overset{M}{\underset{m=1}{\sum }}\sigma_{m}^{2}H_{im}\left(f\right)H_{jm}^{*}\left(f\right),\\ P_{ij}\left(f\right)=\overset{M}{\underset{m=1}{\sum }}\frac{1}{\sigma_{m}^{2}}{\overset{̅}{A}}_{mi}^{*}\left(f\right){\overset{̅}{A}}_{mj}\left(f\right). \end{array}$$

The spectral decompositions in (9) lead to decompose the Coh and PCoh defined in (6a) and (6b) as:

(10a) $$\begin{array}{c} \Gamma_{ij}\left(f\right)=\overset{M}{\underset{m=1}{\sum }}\frac{\sigma_{m}H_{im}\left(f\right)}{\sqrt{S_{ii}\left(f\right)}}\frac{\sigma_{m}H_{jm}^{*}\left(f\right)}{\sqrt{S_{jj}\left(f\right)}}=\overset{M}{\underset{m=1}{\sum }}\gamma_{im}\left(f\right)\gamma_{jm}^{*}\left(f\right), \end{array}$$

(10b) $$\begin{array}{c} \begin{array}{cc} \Pi_{ij}\left(f\right) & =-\overset{M}{\underset{m=1}{\sum }}\frac{\left(1/\sigma_{m}\right){\overset{̅}{A}}_{mj}\left(f\right)}{\sqrt{P_{jj}\left(f\right)}}\frac{\left(1/\sigma_{m}\right){\overset{̅}{A}}_{mi}^{*}\left(f\right)}{\sqrt{P_{ii}\left(f\right)}}\\ & =-\overset{M}{\underset{m=1}{\sum }}\pi_{mj}\left(f\right)\pi_{mi}^{*}\left(f\right). \end{array} \end{array}$$

The last terms in (10a) and (10b) contain, respectively, the so-called *directed coherence* (DC) and *partial directed coherence* (PDC), which we define in this study as:

(11a) $$\begin{array}{c} \gamma_{ij}\left(f\right)=\frac{\sigma_{j}H_{ij}\left(f\right)}{\sqrt{\sum_{m=1}^{M}\sigma_{m}^{2}{\left|H_{im}\left(f\right)\right|}^{2}}}, \end{array}$$

(11b) $$\begin{array}{c} \pi_{ij}\left(f\right)=\frac{\left(1/\sigma_{i}\right){\overset{̅}{A}}_{ij}\left(f\right)}{\sqrt{\sum_{m=1}^{M}\left(1/\sigma_{m}^{2}\right){\left|{\overset{̅}{A}}_{mj}\left(f\right)\right|}^{2}}}. \end{array}$$

The DC as defined in (11a) was originally proposed by Saito and Harashima [46], and further developed as connectivity measure by Baccala et al. [18]. Note that the directed transfer function (DTF) defined in [12] is a particularization of the DC in which the input variances are all equal (*σ* ^2^_1_ = *σ* ^2^_2_ = ⋯ = *σ* ^2^_*M*_) so that they cancel each other in (11a). The quantity which we define as PDC in (11b) was named “generalized PDC” in [20], while the original version of the PDC [19] was not including inner normalization by the input noise variances; our definition (11b) follows directly from the decomposition in (10b). We note that other variants of the PDC estimator have been recently provided: the “information PDC” [21], which under the hypothesis of strict causality reduces to (11a), has been proposed as a measure bridging frequency and information domains; the “renormalized PDC” [22] has been proposed to allow drawing conclusion about the interaction strength by normalization. Here, besides the meaningful dual derivation of DC and PDC as factors in the decomposition of Coh and PCoh, we further justify the utilization of the measures defined in (11a) and (11b) noting that they satisfy the desirable property of scale-invariance. On the contrary, as shown by Winterhalder et al. [11], false detections of causality may occur from low variance process to processes with significantly higher variance when the original DTF and PDC estimators are used.

The quantities *γ*_*ij*_(*f*) and *π*_*ij*_(*f*) defined in (11a) and (11b) can be interpreted as measures of the influence of *y*_*j*_ onto *y*_*i*_, as opposed to *γ*_*ji*_(*f*) and *π*_*ji*_(*f*) which measure the interaction occurring over the opposite direction from *y*_*i*_ onto *y*_*j*_. Therefore, the DC and the PDC, being factors in the decomposition of Coh and PCoh, are asymmetric connectivity measures which elicit the directional information from the two symmetric measures. More detailed interpretation of all these measures is provided in the next subsection.

### 3.2. Interpretation

A straightforward interpretation of the four connectivity measures above presented may be obtained considering that they reflect in the frequency domain the different time-domain definitions of connectivity given in Section 2.2. First, we note that the PDC is a measure of direct causality, because the numerator of (11b) contains, with *i* ≠ *j*, the term ${\overset{̅}{A}}_{ij}\left(f\right)$, which is nonzero only when *a*_*ij*_(*k*) ≠ 0 for some *k* and is uniformly zero when *a*_*ij*_(*k*) = 0 for each *k*. Considering the DC, one can show that, expanding $H(f)=\overset{̅}{A}{\left(f\right)}^{-1}$ as a geometric series [36], the transfer function *H*_*ij*_(*f*) contains a sum of terms each one related to one of the (direct or indirect) transfer paths connecting *y*_*j*_ to *y*_*i*_; therefore, the numerator of (11a) is nonzero whenever any path connecting *y*_*j*_ to *y*_*i*_ is significant, that is, when causality occurs from *y*_*j*_ to *y*_*i*_. As to the coupling definitions, we note from (10a) and (10b) that Γ_*ij*_(*f*) ≠ 0 when both *γ*_*im*_(*f*) ≠ 0 and *γ*_*jm*_(*f*) ≠ 0, and Π_*ij*_(*f*) ≠ 0 when both *π*_*mi*_(*f*) ≠ 0 and *π*_*mj*_(*f*) ≠ 0; this suggests that Coh and PCoh reflect respectively coupling and direct coupling relations in accordance with a frequency-domain representation of the definitions given in Section 2. However, (10a) and (10b) explain also the rationale of introducing a mathematical formalism to define spurious direct coupling and spurious coupling. In fact, the fulfillment of Π_*ij*_(*f*) ≠ 0 or Γ_*ij*_(*f*) ≠ 0 at a given frequency *f* is not a sufficient condition for the existence of direct coupling or coupling at that frequency, because the observed relation can be also spurious. The correspondence with the time-domain connectivity definitions and the frequency-domain measures is summarized in Table 1.

The four connectivity measures defined in (6a), (6b), (11a), and (11b) are complex-valued. In order to have real-valued measures, the squared modulus of Coh, PCoh, DC, and PDC is commonly used to measure connectivity in the frequency domain. Therefore, |Γ_*ij*_(*f*)|^2^, |Π_*ij*_(*f*)|^2^, |*γ*_*ij*_(*f*)|^2^, and |*π*_*ij*_(*f*)|^2^ are computed to quantify, respectively, coupling, direct coupling, causality, and direct causality as a function of frequency. All these squared measures are normalized so that they take values between 0, representing absence of connectivity, and 1, representing full connectivity between the processes *y*_*i*_ and *y*_*j*_ at the frequency *f*. This property allows to interpret the value of each squared index as a measure of the “strength” of connectivity. While this interpretation is meaningful for Coh and DC, it is less useful for PCoh and PDC. Indeed, Coh and DC are defined from the elements of the spectral matrix **S**(*f*) (see (6a)–(11a)) and, as such, are easy to interpret in terms of power spectral density. On the contrary, PCoh and PDC are obtained from a dual representation evidencing the inverse spectral matrix **P**(*f*) (see (6b)–(11b)), which is less easy to interpret because inverse spectra do not have a clear physical meaning. On the other hand, PCoh and PDC are useful when one is interested in determining the frequency-domain connectivity structure of a vector process, as they elicit direct connections between two processes in the MV representation. From this point of view, Coh and DC are more confusing as they measure the “total” connectivity between two processes, mixing together direct and indirect effects.

Further interpretation of the directional measures of connectivity, is provided considering the normalization properties ∑_*m*=1_^*M*^|*γ*_*im*_(*f*)|^2^ = 1 and ∑_*m*=1_^*M*^|*π*_*mj*_(*f*)|^2^ = 1, indicating that the DC is normalized with respect to the structure that receives the signal and the PDC is normalized with respect to the structure that sends the signal [19]. Combined with (9), these properties lead to represent the spectra and inverse spectra of a scalar process, that is, the diagonal elements of **S**(*f*) and **P**(*f*), as:

(12a) $$\begin{array}{c} S_{ii}\left(f\right)=\overset{M}{\underset{m=1}{\sum }}S_{i|m}\left(f\right),\;\;S_{i|m}\left(f\right)={\left|\gamma_{im}\left(f\right)\right|}^{2}S_{ii}\left(f\right), \end{array}$$

(12b) $$\begin{array}{c} P_{jj}\left(f\right)=\overset{M}{\underset{m=1}{\sum }}P_{j\to m}\left(f\right),\;\;P_{j\to m}\left(f\right)={\left|\pi_{mj}\left(f\right)\right|}^{2}P_{jj}\left(f\right), \end{array}$$

where *S*_*i*|*m*_(*f*) is the part of *S*_*ii*_(*f*) due to *y*_*m*_, and *P*_*j*→*m*_(*f*) is the part of *P*_*jj*_(*f*) directed to *y*_*m*_. Thus, the DC and the PDC may be viewed as the relative amount of power of the output process which is received from the input process, and the relative amount of inverse power of the input process which is sent to the output process, respectively. Again, inverse power quantifies direct causality but is of difficult physical interpretation, while power is straightforward to interpret but includes both direct and indirect effects. Therefore, a desirable development would be to split the DC into direct and indirect contributions, in order to exploit the advantages of both representations. However, such a development is not trivial, as recently shown by Gigi and Tangirala [32] who elicited the presence of an interference term which prevents the separation of direct and indirect energy transfer between two variables of a MV process.

**Figure 3:**
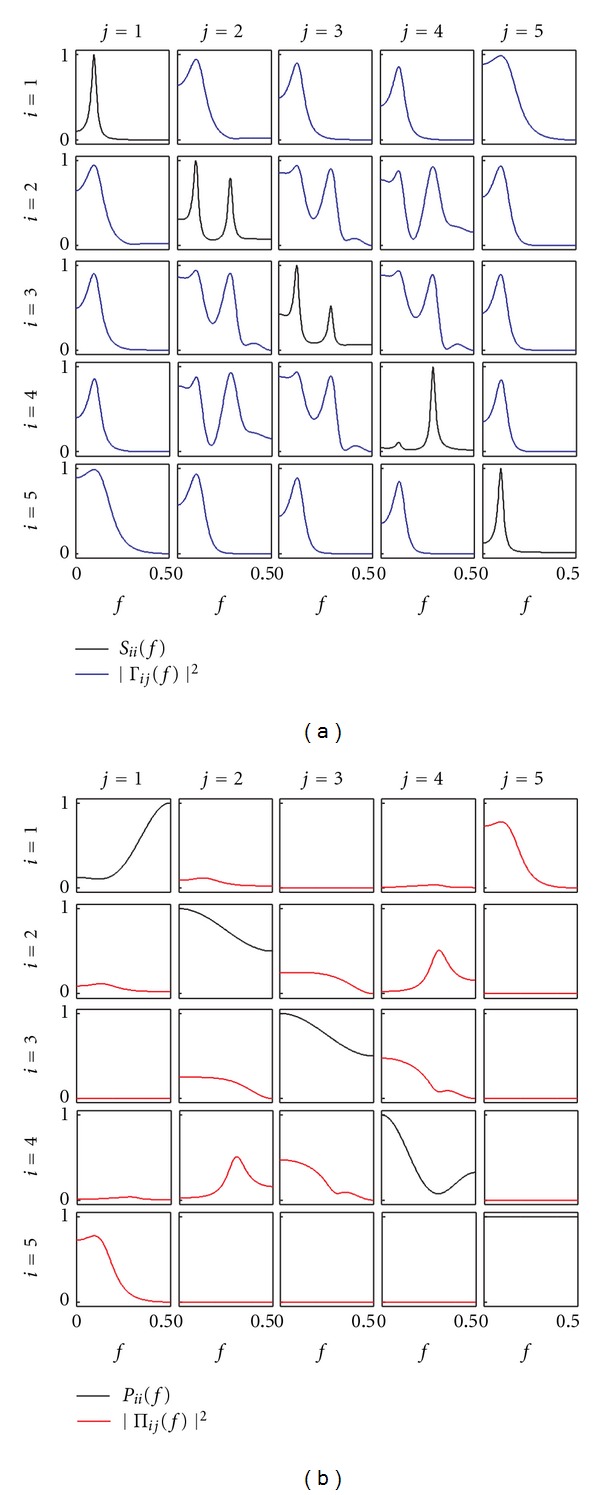
Spectral functions and frequency domain measures of coupling for the illustrative MVAR process of (4). (a) Power spectral density of the process *y*_*i*_ (*S*_*ii*_(*f*), black) and coherence between *y*_*i*_ and *y*_*j*_ (|Γ_*ij*_(*f*)|^2^, blue). (b) Inverse power spectral density of *y*_*i*_ (*P*_*ii*_(*f*), black) and partial coherence between *y*_*i*_ and *y*_*j*_ (|Π_*ij*_(*f*)|^2^, red).

To compare the behavior of the presented connectivity measures and to discuss their properties, let us consider the frequency-domain representation of the theoretical example with time-domain representation given by (4). The trends of spectral and cross-spectral density functions are depicted in Figure 3. The spectra of the five processes, reported as diagonal plots in Figure 3(a), exhibit clear peaks at the frequency of the two oscillations imposed at ~0.1 Hz and ~0.3 Hz for *y*_1_ and *y*_4_, respectively, and appear also in the spectra of the remaining processes according to the imposed causal information transfer. On the contrary the inverse spectra, reported as diagonal plots in Figure 3(b), do not provide clear information about such an oscillatory activity. Off-diagonal plots of Figures 3(a) and 3(b) depict, respectively, the squared magnitudes of Coh and PCoh; note the symmetry of the two functions (Γ_*ij*_(*f*) = Γ_ji_*(*f*), Π_*ij*_(*f*) = Π_ji_*(*f*)), reflecting the fact that they cannot account for directionality of the considered interaction. The comparison of Figure 3(a) with Figure 1(d), and of Figure 3(b) with Figure 1(c), evidences how Coh and PCoh provide a spectral representation of coupling and direct coupling: indeed, the frequency-domain functions are uniformly zero when the corresponding connectivity relation is absent in the time domain. Note that the spurious direct coupling and spurious coupling connections evidenced in Figures 1(c) and 1(d) cannot be pointed out from the frequency-domain representations of Figure 3: for example, the coherence between *y*_5_ and all other processes is very high at ~0.1 Hz although only the coupling *y*_1_⇔*y*_5_ is not spurious. Another observation regards interpretability of the absolute values: while Coh shows clear peaks at the frequency of coupled oscillations (~0.1 Hz and ~0.3 Hz) when relevant, PCoh is less easy to interpret as sometimes the squared modulus does not exhibit clear peaks (e.g., |Π_23_(*f*)|^2^, |Π_34_(*f*)|^2^) or is very low (e.g., |Π_12_(*f*)|^2^, |Π_14_(*f*)|^2^).

**Figure 4:**
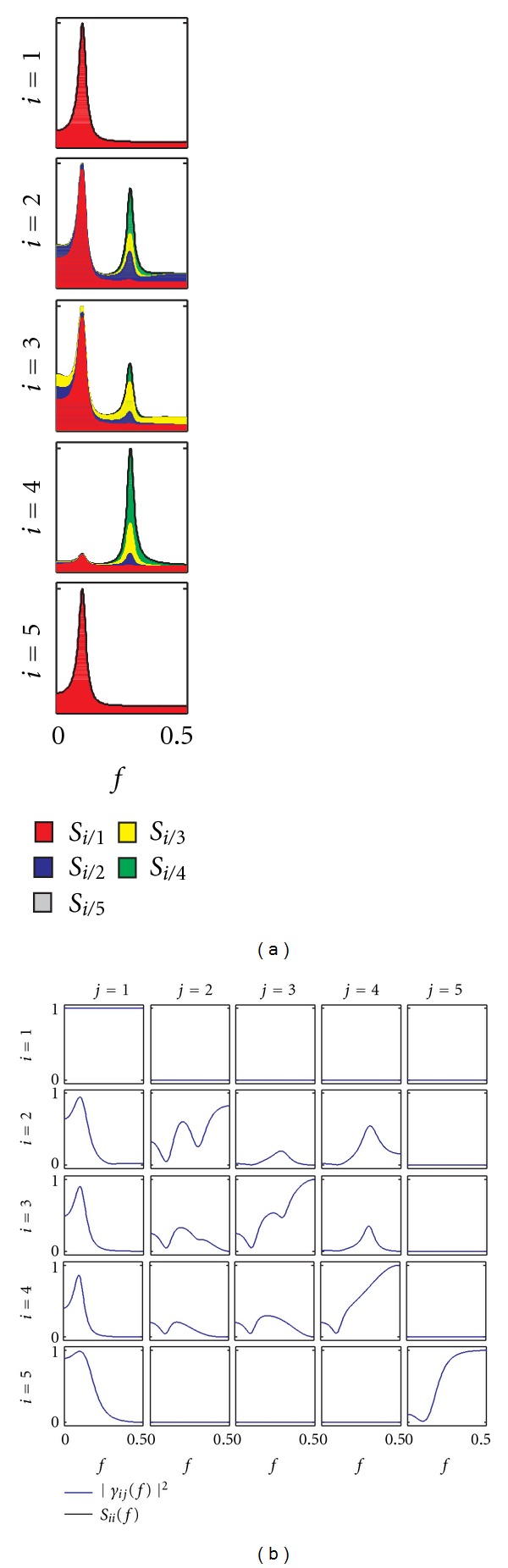
Decomposition of the power spectrum of each process *y*_*i*_ in (4), *S*_*ii*_(*f*), into contributions coming from each process *y*_*j*_ (*S*_*i*|*j*_, shaded areas in each plot) (a), and corresponding squared DC from *y*_*j*_ to *y*_*i*_, |*γ*_*ij*_(*f*)|^2^ (b) depicted for each *i*, *j* = 1,…, *M*.

Figure 4 depicts the spectral decomposition of the MVAR process, as well as the trends resulting for the DC function from this decomposition. Note that the DC reflects the pattern of causality depicted in Figure 1(b), being uniformly zero along all directions over which no causality is imposed in the time domain.  Figure 4(a) provides a graphical representation of (12a), showing how the spectrum of each process can be decomposed into power contributions related to all processes; normalizing these contributions, one gets the squared modulus of the DC, as depicted in Figure 4(b). In the example, the spectrum of *y*_1_ is decomposed in one part only, deriving from the same process. This indicates that no part of the power of *y*_1_ is due to the other processes. The absence of external contributions is reflected by the null profiles of the squared DCs |*γ*_1*j*_(*f*)|^2^ for each *j* > 1, which also entail a flat unitary profile for |*γ*_11_(*f*)|^2^. On the contrary, the decompositions of *y*_*i*_, with *i* > 1, results in contributions from the other processes, so that the squared DC |*γ*_*ij*_(*f*)|^2^ is nonzero for some *j* ≠ *i*, and the squared DC |*γ*_*ii*_(*f*)|^2^ is not uniformly equal to 1 as a result of the normalization condition. In particular, we note that the power of the peak at *f*_1_ = 0.1 Hz is due to *y*_1_ for all processes (red areas in Figure 4(a)), determining very high values of the squared DC in the first column of the matrix plot in Figure 3(b), that is, |*γ*_*i*1_(*f*_1_)|^2^ ≈ 1; this behavior represents in the frequency domain the causality relations imposed from *y*_1_ to all other processes. Note that, as a consequence of the normalization condition of the DC, the high values measured at ~0.1 Hz for |*γ*_*i*1_|^2^ entail very low values, at the same frequency, for |*γ*_*ij*_|^2^ computed with *j* > 1. Whereas this property is straightforward when the studied effect is direct, in the case of indirect causality, it suggests that the DC modulus tends to ascribe the measured causal coupling to the source process (i.e., the first process) rather than to the intermediate processes of the considered cascade interaction. The remaining causality relations are relevant to the oscillation at *f*_2_ = 0.3 Hz, which is generated in *y*_4_ and then re-transmitted to the same process through a loop involving *y*_2_ and *y*_3_. This loop of directed interactions is reflected by the presence of a peak at ~0.3 Hz of the spectra of *y*_2_, *y*_3_, and *y*_4_, as well as by the spectral decomposition within this frequency band (Figure 4(a)). This decomposition results, after proper normalization, in the nonzero DCs |*γ*_42_|^2^, |*γ*_23_|^2^, |*γ*_34_|^2^ (direct causality) and |*γ*_43_|^2^, |*γ*_32_|^2^, |*γ*_24_|^2^ (indirect causality) observed at *f*_2_.

**Figure 5:**
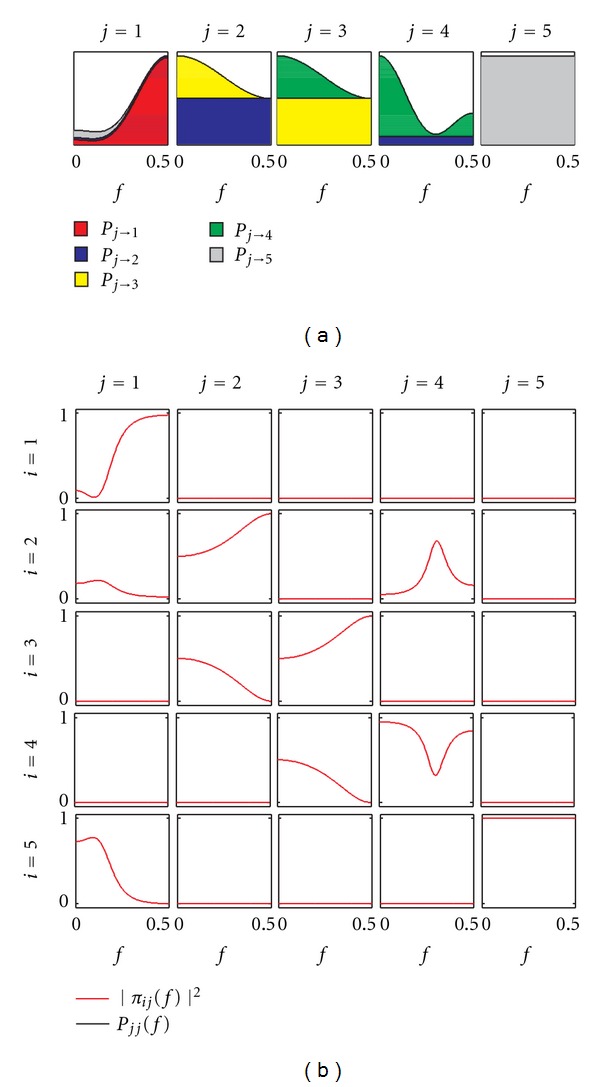
Decomposition of the inverse power spectrum of each process *y*_*i*_ in (4), *P*_*jj*_(*f*), into contributions directed towards each process *y*_*i*_ (*P*_*j*→*i*_, shaded areas in each plot) (a), and corresponding squared PDC from *y*_*j*_ to *y*_*i*_, |*π*_*ij*_(*f*)|^2^ (b) depicted for each *i*, *j* = 1,…, *M*.

A dual interpretation to that provided above holds for the decomposition of the inverse spectra into absolute and normalized contributions sent to all processes, which are depicted for the considered example in the area plot of Figure 5(a) and in the matrix PDC plot of Figure 5(b), respectively. The difference is that now contributions are measured as outflows instead as inflows, are normalized to the structure sending the signal instead to that receiving the signal, and reflect the concept of direct causality instead that of causality. With reference to the proposed example, we see that the inverse spectrum of *y*_1_ is decomposed into contributions flowing out towards *y*_2_ and *y*_5_ (blue and gray areas underlying *P*_11_(*f*) in Figure 5(a)), which are expressed in normalized units by the squared PDCs |*π*_21_|^2^ and |*π*_51_|^2^. While *y*_2_, *y*_3_, and *y*_5_ interact in a closed loop (absolute units: *P*_2→3_ ≠ 0, *P*_3→4_ ≠ 0, *P*_4→2_ ≠ 0; normalized units: |*π*_32_|^2^ ≠ 0, |*π*_43_|^2^ ≠ 0, |*π*_24_|^2^ ≠ 0), *y*_5_ does not send information to any process (*P*_5→*i*_ = 0, |*π*_*i*5_|^2^ = 0, *i* = 1,2, 3,4). As can be seen comparing Figure 5 with Figure 1(a), the profiles of *P*_*j*→*i*_ and |*π*_*ij*_|^2^ provide a frequency-domain description, respectively, in absolute and normalized terms, of the imposed pattern of direct causality. We note also that all inverse spectra of a process contain a contribution coming from the same process, which describes the part of *P*_*jj*_(*f*) which is not sent to any of the other processes (*P*_*j*→*j*_ in Figure 4(a)). After normalization, this contribution is quantified by the PDC |*π*_*jj*_|^2^, as depicted by the diagonal plots of Figure 5(b).

## 4. Practical Analysis

### 4.1. Model Estimation

The practical application of the theoretical concepts described in this tutorial paper is based on considering the available set of time series measured from the system under analysis, {*y*_*m*_(*n*),*m* = 1,…, *M*; *n* = 1,…*N*}, as a finite-length realization of the MV stochastic process describing the evolution of the system over time. Hence, the descriptive equation (3) is seen as a model of how the observed data have been generated, and model identification algorithms have to be applied for providing estimates of the model coefficients to be used in place of the true unknown coefficients in the generating equations. Obviously, the estimates will never be the exact coefficients, and consequently the frequency-domain measures estimated from the real data will always be an approximation of the true functions. The goodness of the approximation depends on practical factors such as the data length, and on the type and parameters of the procedure adopted for the identification of the model coefficients. Identification of the MVAR model (3) can be performed with relative ease by means of estimation methods based on the principle of minimizing the prediction error, that is, the difference between actual and predicted data (see, e.g., [15] or [47] for detailed descriptions). A simple estimator is the MV least-squares method, which is based first on representing (3) in compact representation as **Y** = **A** **Z** + **U**, where **A** = [**A**(1) ⋯ **A**(*p*)] is the *M* × *pM* matrix of the unknown coefficients, **Y** = [**Y**(*p* + 1) ⋯ **Y**(*N*)] and **U** = [**U**(*p* + 1) ⋯ **U**(*N*)] are *M* × (*N* − *p*) matrices, and **Z** = [**Z**_1_^*T*^⋯**Z**_*p*_^*T*^]^*T*^ is a *pM* × (*N* − *p*) matrix having **Z**_*i*_ = [**Y**(*p* − *i* + 1) ⋯ **Y**(*N*-*i*)] as *i*th row block (*i* = 1,…*p*). The method estimates the coefficient matrices through the well-known least-squares formula: $\hat{A}=YZ^{T}{\left[ZZ^{T}\right]}^{-1}$, and the innovation process as the residual time series: $\hat{U}=\hat{A}Z-Y$. As to model order selection, several criteria exist for its determination [47]. Common approaches are to follow the Akaike information criterion (AIC, [48]) or to the Bayesian information criterion (BIC, [49]), based on setting the order *p* at the value for which the respective figure of merit, (i.e., AIC(*p*) = *N* · log⁡⁡(det⁡Σ) + 2*M* ^2^ *p*, or BIC(*p*) = *N* · log⁡⁡(det⁡Σ) + log⁡⁡(*N*)*M* ^2^ *p*) reaches a minimum within a predefined range of orders. While the model identification and order selection methods presented here have good statistical properties, more accurate approaches exist; for example, we refer the reader to [50] for a comparison of different MVAR estimators, to [51] for order selection criteria optimized for MVAR models, and to [52] for an identification approach combining MVAR coefficient estimation and order selection.

After model identification, validation steps are necessary to guarantee a correct interpretation of the obtained results. Model validation refers to the use of a range of diagnostic tools which are available for checking the adequacy of the estimated structure. In particular, identification of the MVAR model (3) should result in temporally uncorrelated and mutually independent residuals $\hat{U}(n)$. These assumptions may be checked, for example, using the Ljung-Box portmanteau test for whiteness and the Kendall's *τ* test for independence [47]. Mutual independence of the residuals has to be checked particularly at zero lag, because the existence of correlated model innovations violates the assumption of strict causality. Although these tests are often skipped in practical analysis, we stress the importance of performing model validation, because failure to satisfy the model assumptions is a clear indication of model mis-specification (see Section 5 for a more detailed description of this problem).

### 4.2. Statistical Significance of Connectivity Measures

Besides confirming the suitability of the estimated model, another issue of great practical importance is the assessment of the statistical significance of the derived connectivity measures. Due to practical estimation problems, nonzero values are indeed likely to occur at some frequencies even in the case of absence of a true interaction between the two considered processes. This problem is commonly faced by means of statistical hypothesis testing procedures based on setting a threshold for significance at the upper limit of the confidence interval of the considered index, where confidence intervals are based on the sampling distribution of the index computed under the null hypothesis of absence of connectivity. Comparing at each specific frequency the estimated index with the threshold allows rejection or acceptance of the null hypothesis according to the predetermined level of significance. The sampling distribution in the absence of connectivity may be derived either theoretically or empirically: theoretical approaches are elegant and computationally more efficient, empirical ones are more general and free of analytical approximations. While the statistical analytical threshold for the Coh estimator can be found in classic time series analysis books (e.g., [53]), recent theoretical studies have provided rigorous asymptotic distributions for the PDC [54, 55] and its renormalized version [22], as well as for the DC/DTF [36]. As to the determination of empirical significance levels, the most popular approaches consist in applying permutation statistics [56] when the data matrix can be partitioned in many windows from which multiple values of the connectivity measure may be computed, and in applying surrogate data analysis [57] when only one value of the measure is computed. In the latter case, the most followed approach is the generation of the so-called FT surrogate series, which are obtained by a phase randomization procedure applied independently to each series of the considered MV data set. This approach has been proposed to assess the significance of the Coh estimator [58], and has been used also with the causality estimators [17, 59]. A recent development of the FT method is that leading to the generation of the so-called “causal FT” (CFT) surrogates [60]. CFT surrogates were devised specifically for the detection of the significance of causality and direct causality in the frequency domain, and have been shown to outperform FT surrogates as regards the empirical detection of a zero-level for the DC or the PDC [60]. However, it has to be remarked that the computational burden of this new method is significantly larger than that required for the generation of FT surrogates, and this may make very demanding, or even intractable, the assessment of significance when high-dimensional MVAR models are analyzed.

### 4.3. Practical Illustrative Example

In this section, we report a practical application of the presented connectivity analysis to MV neurophysiological time series. Specifically, we considered electroencephalographic (EEG) recordings collected from a subject performing a visuomotor task combining precise grip motor commands with sensory visual feedback. Briefly, the subject was asked to track the variations in size of a square target displayed on a monitor by acting on a pinch grip through his right hand thumb and forefinger. Visual feedback about his performance was provided to the subject by displaying on the monitor another square reflecting the exerted force (the task required to continuously match the two rectangles). EEG signals were acquired (earlobes common reference; sampling rate: 576 Hz) during the experiment according to the standard 10–20 electrode placement enlarged with intermediate positions in scalp areas of interest for the specific task performed. Full details about the experimental protocol can be found in [28].

**Figure 6:**
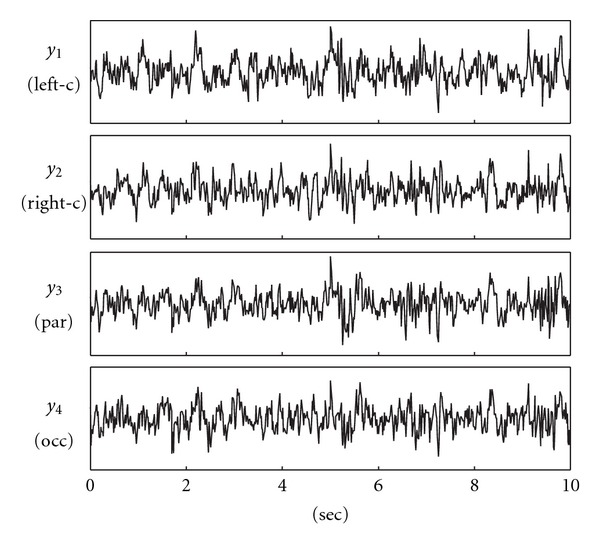
Time series considered for the neurophysiological application: EEG signals recorded at left-central (*y*_1_), right-central (*y*_2_), parietal (*y*_3_), and occipital (*y*_4_) scalp locations during the execution of a visuomotor task (see text for details).

Here, we present the results of frequency-domain connectivity analysis performed for *M* = 4 EEG signals selected as representative of the cortical areas involved in visuomotor integration processes, that is, left and right central areas (motor cortex, electrodes C3 and C4) and posterior-parietal regions (visual area and parietal cortex, electrodes Pz and Oz) [61]. The signals were bandpass filtered (3–45 Hz) to remove power supply noise and extract information about the brain rhythms of interest, and then downsampled to 72 Hz to reduce redundancy. Pre-processed EEGs were carefully inspected to identify possible artifacts, and a stationary window of ten seconds (*N* = 720 samples) was then selected for the analysis. The four analyzed signals are shown in Figure 6.

The coefficients and input covariance of the MVAR model describing the four time series were estimated using the MV least-squares method; the model order, determined as the minimum of the Akaike figure of merit within the range (1,30), was *p* = 8. Model validation was performed checking whiteness and independence of the estimated model residuals by means of the Ljung-Box test and the Kendall test, respectively. The estimated coefficients and input covariance were used to estimate the frequency-domain coupling and causality functions according to (6a), (6b), (11a), and (11b), respectively. Each estimated connectivity function was evaluated inside the beta band of the frequency spectrum (13–30 Hz), in order to investigate connectivity mechanisms related to medium-range interactions among communicating brain areas [28]. The statistical significance of the various connectivity measures computed in the beta band for each specific direction of interaction was assessed by means of an approach based on the generation of surrogate data. The test, which is described in detail in [60], was performed generating a set of 100 surrogate series by means of a phase randomization procedure that preserves the modulus of the Fourier transform of the original series and alters the Fourier phases in a way such that connectivity is destroyed only over the direction of interest; note that the method is specific for each connectivity measure, so that it specifically destroys coupling, direct coupling, causality, or direct causality between two series, respectively, when the significance of Coh, PCoh, DC, or PDC is going to be tested. For each connectivity measure, the threshold for significance was obtained as the 95th percentile (corresponding to 5% significance) of the distribution of the measure computed over the 100 surrogate series.

**Figure 7:**
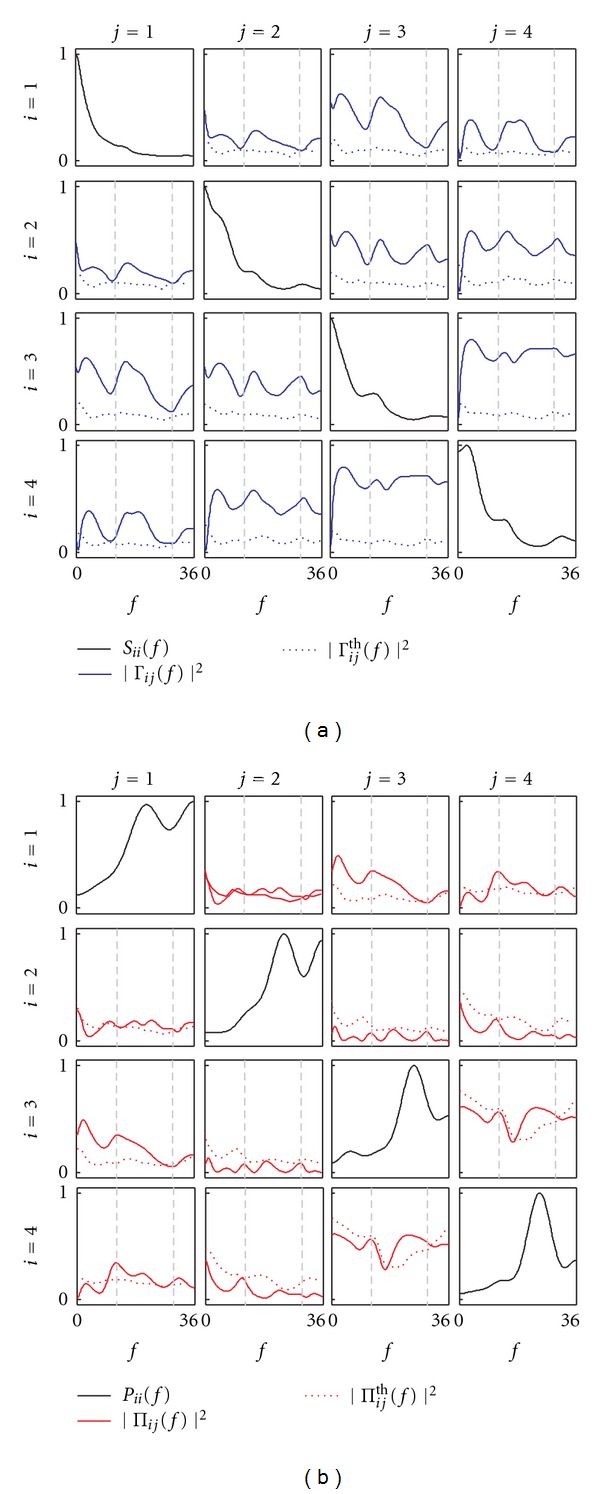
Spectral functions and frequency-domain measures of coupling for the exemplary neurophysiological application (*y*_1_: left central EEG; *y*_2_: right central EEG; *y*_3_: parietal EEG; *y*_4_: occipital EEG). (a), Power spectral density of the process *y*_*i*_(*S*_*ii*_(*f*)) and coherence between *y*_*i*_ and *y*_*j*_ (|Γ_*ij*_(*f*)|^2^) plotted together with its corresponding threshold for significance (|Γ_*ij*_^th^(*f*)|^2^). (b), Inverse power spectral density of *y*_*i*_(*P*_*ii*_(*f*)) and partial coherence between *y*_*i*_ and *y*_*j*_(|Π_*ij*_(*f*)|^2^) plotted together with its corresponding threshold for significance (|Π_*ij*_^th^(*f*)|^2^). Vertical dashed lines in each off-diagonal plot denote the bounds of the beta frequency band (13–30 Hz).

**Figure 8:**
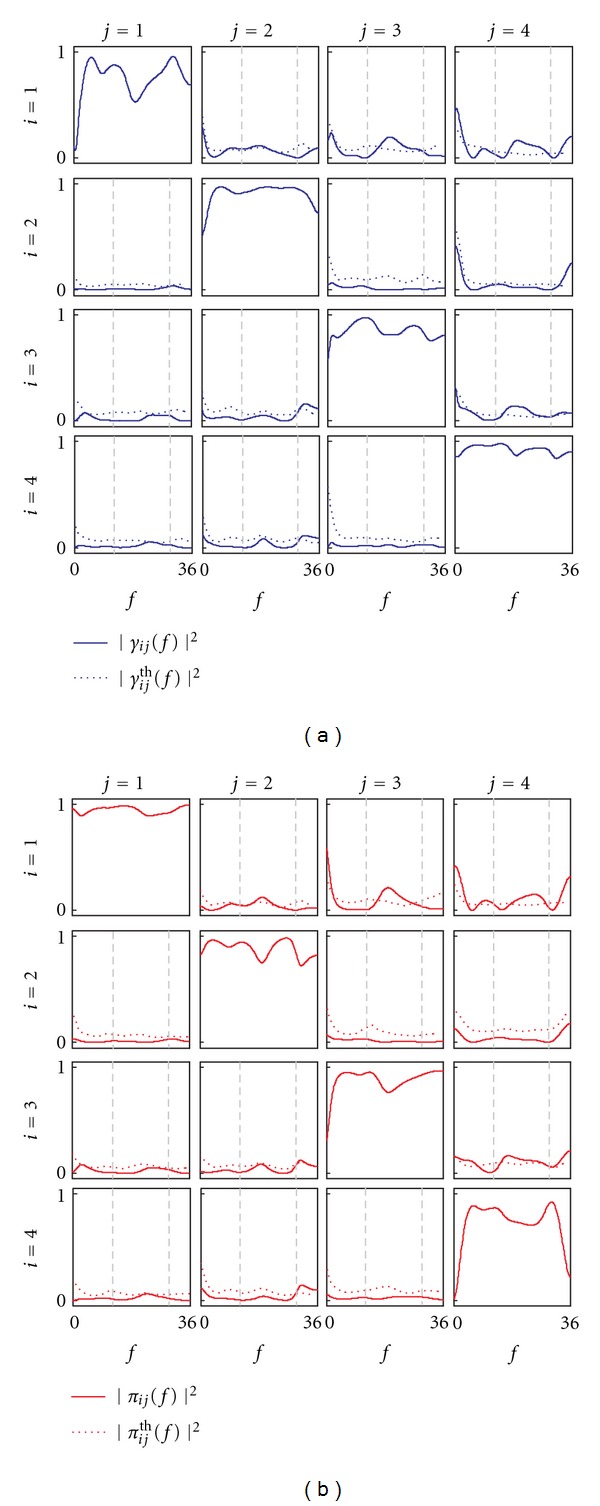
Frequency-domain measures of causality for the exemplary neurophysiological application (*y*_1_: left central EEG; *y*_2_: right central EEG; *y*_3_: parietal EEG; *y*_4_: occipital EEG). (a) Squared DC from *y*_*j*_ to *y*_*i*_, (|*γ*_*ij*_(*f*)|^2^) plotted together with its corresponding threshold for significance (|*γ*_*ij*_^th^(*f*)|^2^). (b) Squared PDC from *y*_*j*_ to *y*_*i*_, (|*π*_*ij*_(*f*)|^2^) plotted together with its corresponding threshold for significance (|*π*_*ij*_^th^(*f*)|^2^). Vertical dashed lines in each off-diagonal plot denote the bounds of the beta frequency band (13–30 Hz).

The results of the analysis are reported in Figures 7 and 8 for coupling and causality measures, respectively. The analysis of coupling indicates that the network of the four interacting signals is fully connected inside the beta band, as documented by the Coh values exceeding the significance threshold for each pair of time series (Figure 7(a)). When this information is particularized to the study of direct coupling through the PCoh, we observe that direct connections are set in the beta band between *y*_3_ and *y*_4_, *y*_1_ and *y*_3_, *y*_1_ and *y*_4_, and *y*_1_ and *y*_2_ (though the PCoh exceeds the significance threshold only slightly in this last case). This suggests a major involvement of the left hemisphere in the connectivity network activated by the visuomotor task, likely due to the dominant role of the left-motor cortical area (signal *y*_1_, electrode C3); the EEG recorded from this area, which is contra-lateral to the moving right hand, is mainly linked to that recorded from the parietal (signal *y*_3_, electrode Pz) and occipital (signal *y*_4_, electrode Oz) areas which are expected to reflect processing of the visual information. The causality analysis depicted in Figure 8 shows how the information about the direction of interaction may be elicited for this application. In particular, the peaks shown inside the beta band by the squared DC computed from *y*_*j*_ to *y*_1_ (*j* = 2,3, 4; first row plots of Figure 8(a)), which are small but exceed the threshold for significance, indicate that a significant part of the power spectrum of *y*_1_ is due to the other channels. Considering also the significant DC from *y*_4_ to *y*_3_, we can infer the presence of a non negligible information transfer from the occipital to the left-central cortical regions. This finding is confirmed by the analysis of direct causality performed through the PDC (Figure 8(b)), which indicates that both the direct pathway *y*_4_ → *y*_1_ and the indirect pathway *y*_4_ → *y*_3_ → *y*_1_ are active in determining causality from the occipital to the left-central areas, as documented by the significant values of |*π*_14_|^2^, |*π*_34_|^2^, and |*π*_13_|^2^ inside the beta band. The unidirectional nature of the information flow is confirmed by the fact that both the DC (Figure 8(a)) and the PDC (Figure 8(b)) resulted non significant over all directions from *y*_*j*_ to *y*_*i*_ with *j* < *i*. Taken together, all these results suggest the existence of a functional link between motor and visual cortices during the performed visuomotor task, and lead to hypothesize an active role of the visual feedback in driving the beta oscillations measured in the motor cortex. A full analysis of this experiment, performed on more subjects and leading to a deeper interpretation of the involved sensorimotor integration mechanisms, is reported in [62].

## 5. Limitations and Challenges

In spite of its demonstrated usefulness and widespread utilization, MVAR-based connectivity analysis is often challenged by a number of issues that need to be taken into serious account to avoid an improper utilization of this tool. A key issue in this regard is that of model mis-specification, which occurs when the developed MVAR model does not adequately capture the correlation structure of the observed dataset. There are several factors which may determine model mis-specification, including utilization of an inappropriate model structure, incorrect model order selection, effects of non-modeled latent variables, and aspects not accountable by the traditional MVAR structure such as nonstationarity and nonlinearity. Most of these factors are typically reflected in the structure of the model residuals, resulting in a failure for the model to fulfill the assumptions of whiteness and independence of the innovations (see Section 4.1). When the MVAR model is mis-specified, utilization of the related frequency-domain connectivity measures is potentially dangerous and is generally not recommended, because it may lead to infer misleading or inconsistent connectivity patterns, and thus to erroneously interpret the physiological mechanism under investigation. In the following, we discuss the limitations posed on MVAR-based connectivity analysis by each of the factors listed above, and we outline recent work that may address, at least in part, the related pitfalls.

### 5.1. Appropriateness of Model Structure

A common cause for model mis-specification is the inadequacy of the MVAR model structure to fully describe the observed set of MV time series. Validation tests (see Section 4.1) provide objective criteria on whether the model has the capability of resolving the measured dynamics and dynamical interactions. The requirements of whiteness and independence of the model residuals can be understood considering that, if the model has captured the whole temporal structure of the data, what remains after modeling (i.e., the residuals) has no temporal structure. Failure of fulfilling the white noise assumption means that the spectral properties of the signals are not fully described by the autoregression so that, for example, important power amounts in specific frequency bands could not be properly quantified. When the whiteness test is not passed, the experimenter should consider moving to different model structures, such as MV dynamic adjustment forms having the general structure of MVAR networks fed by individual colored AR noises at the level of each signal [37, 38].

Failure of fulfilling the requirement of mutual independence of the residuals corresponds to violating the assumption of strict causality of the MVAR process. This is an indication of the presence of significant instantaneous causality, and occurs anytime the time resolution of the measurements is lower than the time scale of the lagged causal influences occurring among the observed series. This situation is common in the analysis of neural data, such as fMRI where the slow dynamics of the available signals make rapid causal influences appearing as instantaneous, or EEG/MEG where instantaneous effects are likely related to signal cross-talk due to volume conduction [56]. In this case, the spectral decompositions leading to the definition of DC and PDC do not hold anymore, and this may lead to the estimation of erroneous frequency-domain connectivity patterns like spurious nonzero DC and PDC profiles indicating causality or direct causality for connections that are actually absent [29, 63]. A possible solution to this problem is to use a higher sampling rate, but this would increase the data size and—most important—would introduce redundancy that might hamper model identification [64]. A recently proposed approach is to incorporate zero-lag interactions in the MVAR model, so that both instantaneous and lagged effects are described in terms of the model coefficients and may be described in the frequency domain [29, 63, 65]. This approach is very promising but introduces non-trivial identification issues which could limit its practical utilization. In fact, ordinary least-squares identification, though recently proposed for identification of the extended model [65], is not feasible because it forces arbitrarily the solution; to guarantee identifiability without prior constraints, additional assumptions such as non-gaussianity of the residuals have to be posed [63, 66].

### 5.2. Model Order Selection

Even when the most suitable model structure is selected for describing the available MV dataset, model mis-specification may still occur as a consequence of an inappropriate selection of the model order. Model order selection is in fact an issue in real data analyses where the true order is usually unknown. In general, a too low model order would result in the inability to describe essential information about the MV process, while a too high-order would bring about overfitting effects implying that noise is captured in the model together with the searched information. Therefore, a tradeoff needs to be reached between good data representation and reasonably low model complexity. While information criteria like AIC or BIC are very popular (see also Section 4.2), a correct model order assessment is rather difficult because the estimated order may not meet the user expectations (in terms of spectral resolution when it is too low, or in terms of interpretability of highly variable spectral profiles when it is too high), or may even remain undetermined as the AIC/BIC figures of merit do not reach a clear minimum within the range searched [25, 29, 67]. Simulation studies have shown that both underestimation and overestimation of the correct model order may have serious implications on connectivity analysis, with an increasing probability of missed and false-positive connections [68, 69]. A recent interesting result is the apparent asymmetry in the adverse effects on connectivity analysis of choosing a wrong model order, with more severe effects for underestimation than overestimation [69], which suggests to prefer higher orders while tuning this parameter in practical MVAR analysis. Adopted with the proper cautiousness, this choice would be good as it is also known to increase frequency resolution of connectivity estimates and to favor the achievement of whiteness for the model residuals.

### 5.3. Selection of Variables

The tools surveyed in this study to measure connectivity are fully multivariate, in the sense that they are based on MVAR analysis whereby all the considered time series (often more than two) are modeled simultaneously. This approach overcomes the known problems of repeated bivariate analysis applied to multiple time series, consisting, for example, in the detection of false coupling or causality between two series when they are both influenced by a third series [13, 70]. Nevertheless, in practical experimental data analysis, it is often not possible to have access to the complete set of variables which are relevant to the description of the physiological phenomena of interest. This issue goes back to the requirement of completeness of information stated for causality analysis [40], and raises the problem that unmeasured latent variables—often called unobserved confounders—can lead to detection of apparent connectivity patterns that are actually spurious, even when multivariate tools are at hand. Dealing with latent variables seems a daunting challenge, because there is no unique way to determine the information set relevant for a given problem. However, recent developments have started giving a response to this challenge through the proposition of approaches to causality analysis based on the idea that latent variables may give rise to zero-lag correlations between the available modeled series, and thus can be uncovered, at least in part, by further analyzing such a correlation [71].

A different but related problem to that of completeness is the redundancy in the group of the selected variables. Historically, the issue of redundant variables has been viewed more as a problem of increased model complexity and related decrease of parameter estimation accuracy in the modeling of massively MV data sets (such as those commonly recorded in fMRI or high resolution EEG-MEG studies). This problem has been tackled through the introduction of network reduction approaches (e.g., [72]) or sparse (regularized/penalized) regression techniques (e.g., [73]), which allow to perform efficient high-dimensional MVAR analysis. More recent works have introduced a general formalism to recognize redundant variables in time series ensembles, showing that the presence of redundant variables affects standard connectivity analyses, for example, leading to underestimation of causalities [74, 75]. An elegant solution to this problem, proposed in [74], consists in performing a block-wise approach whereby redundancy is reduced grouping the variables in a way such that a properly defined measure of total causality is maximized.

### 5.4. Nonstationarity and Nonlinearity

The MVAR-based framework for connectivity analysis is grounded on the basic requirement that the set of observed multiple time series is suitably described as a realization of a vector stochastic process which is both linear and stationary. Despite this, nonlinear and nonstationary phenomena are abundant in physiological systems, and it is well known that MVAR-model analysis performed or nonlinear and/or nonstationary data may lead to a range of erroneous results [76]. In general, nonlinear methods are necessary to perform a thorough evaluation of connectivity whenever nonlinear dynamics are expected to determine to a non-negligible extent the evolution over time of the investigated time series. Analogously, when nonstationary data are expected to reflect connectivity patterns exhibiting physiologically relevant changes over time, it makes sense to use time-varying methods for the detection of coupling or causality. In these situations, several nonlinear/nonstationary time series analysis approaches may be pursued. Nonlinear methods range from local linear MVAR models exploited to perform local nonlinear prediction [6, 77] to nonlinear kernels [5] and to model-free approaches based on information theory [7, 78], phase synchronization [79], and state-space interdependence analysis [80]. As to nonstationary analysis, one simple approach is to study short-time windows which may be taken as locally stationary [67], while more complex but potentially more efficient approaches include spectral factorization of wavelet transforms [81] and the combination of state space modeling and time-dependent MVAR coefficients [82].

On the other hand, linear time-invariant analyses like the MVAR-based approach presented in this study remains of great appeal for the study of physiological interactions, mainly because of their simplicity, well-grounded theoretical basis, and shorter demand for data length in practical analysis. The problem of non-stationarity may be dealt with following common practical solutions like, for example, looking for analysis windows in which the recorded signals are stable and satisfy stationarity tests, and filtering or differentiating the data if necessary (though this has to be done cautiously [83]). The problem of nonlinearity may be faced looking for experimental setups/conditions in which the system dynamics may be supposed as operating, at the level of the recorded signals, according to linear mechanisms. In fact, based on the observation that nonlinear systems often display extensive linear regimes, many neuroscience studies have shown that the linear approximation may suffice for describing neurophysiological interactions, especially at a large-scale level (see, e.g., the reviews [56, 84]). Even in circumstances where nonlinear behaviors are manifest, such as simulated chaotic systems or real EEG activity during certain phases of epileptic seizure, linear techniques have been shown to work reasonably well for the detection of connectivity patterns [11, 85]. In addition, we remark that the large majority of nonlinear methods used in the MV analysis of neurophysiological signals do not provide specific frequency-domain information [2]. The existing bivariate nonlinear frequency-domain tools, such as cross-bispectrum and cross-bicoherence [86], are useful to characterize dependencies between oscillations occurring in different frequency bands. However, analysis of coupling and causality between iso-frequency rhythms observed in different signals is intrinsically linear, and this further supports utilization of the linear framework for this kind of analysis.

## 6. Conclusions

In this tutorial paper, we have illustrated the theoretical interpretation of the most common frequency-domain measures of connectivity which may be derived from the parametric representation of MV time series, that is, Coh, PCoh, DC, and PDC. We have shown that each of these four measures reflects in the frequency domain a specific time-domain definition of connectivity (see Table 1). In particular, while Coh and PCoh are symmetric measures reflecting the coupling between two processes, they can be decomposed into non-symmetric factors eliciting the directional information from one process to another, these factors being exactly the DC and the PDC. Moreover, PCoh and PDC measure direct connectivity between two processes, while Coh and DC account for both direct and indirect connections between two processes in the MV representation.

We have pointed out the existence of a dual description of the joint properties of an MVAR process such that Coh and DC on one side, and PCoh and PDC on the other side, may be derived from the spectral matrix describing the process and from its inverse, respectively. This duality relationship highlights advantages and disadvantages of the various connectivity measures. Being related to spectral densities, Coh and DC provide meaningful quantification of coupling and causality in terms of (normalized) power shared by the two considered processes; on the contrary, PCoh and PDC are derived through an analysis performed in the inverse spectral domain which cannot provide evident physical information for the absolute values of the resulting indexes. On the other side, the procedure of “partialization” implicit in the computation of the inverse spectral matrix lets PCoh and PDC elicit the structural information of the MV process, so that they reflect direct connections only; this ability is not shared by Coh and DC, which mix together direct and indirect transfer pathways and thus cannot provide a straightforward representation of the connectivity structure of the process. Another interesting observation comes from the decomposition of Coh (or, dually, of PCoh) between two processes into DC (or PDC) terms involving a third process (10a) and (10b); these relationships indicate that spurious (direct) coupling may be detected when the two processes under analysis, though not being truly connected, are connected to another common process.

The picture emerging from these results provides suggestions for the utilization of the various connectivity measures in the analysis of MV processes. First, measures of causality should be preferred to measures of coupling, as the latter cannot provide directional information and may be confusing as they are sensitive to spurious connectivity. Second, both DC and PDC should be considered as causality measures because they complement each other in terms of advantages and drawbacks: DC measures causality in meaningful physical terms as power contributions, but cannot separate direct effects from indirect ones; PDC determines the correct interaction structure in terms of direct causal effects, but its absolute values lack of straightforward interpretability. As to recommendations for the practical analysis of real MV time series, we remark the importance of validation tests, which constitute important safeguards against drawing erroneous inferences consequently to model misspecification, and of assessing the significance of each estimated connectivity measure, which is fundamental to provide statistical validity to the estimated MV process structure. Taking all these aspects into account, we have shown the practical applicability of the presented frequency-domain connectivity measures in neurophysiology. In the reported example, the simultaneous computation of Coh, PCoh, DC, and PDC, and of their specific significance thresholds, from multiple EEG recorded during the execution of a combined visuomotor task led us to infer the existence of a specific network subserving sensorimotor integration. This network was characterized by a significant coupling between visual and motor cortical regions, which was particularized into significant causality from the occipital to the left central cortex, suggesting a driving role of the visual feedback on the EEG activity of the motor areas.

## Figures and Tables

**Table 1:** Connectivity definitions and conditions for their existence. (See text for details).

Definition		MV closed-loop process	MVAR process, time domain	MVAR process, Frequency domain
Direct causality	*y*_*j*_ → *y*_*i*_	Knowledge of *Y*_*j*_ improves prediction of *y*_*i*_ (*n*)	*a*_*ij*_(*k*) ≠ 0	**π*_ij_*(*f*) ≠ 0

Causality	*y*_*j*_⇒*y*_*i*_	*y*_*j*_ → *y*_*m*_ ⋯ →*y*_*i*_	*a*_*mj*_(*k*) ≠ 0,…, *a*_*im*_(*k*) ≠ 0	**γ*_ij_*(*f*) ≠ 0

Direct coupling	*y*_*i*_↔*y*_*j*_	*y*_*i*_ → *y*_*j*_ or *y*_*j*_ → *y*_*i*_	*a*_*ji*_(*k*) ≠ 0 or *a*_*ij*_(*k*) ≠ 0	Π*_ij_*(*f*) ≠ 0
Spurious direct coupling		*y*_*i*_ → *y*_*m*_ and *y*_*j*_ → *y*_*m*_	*a*_*mi*_(*k*) ≠ 0 and *a*_*mj*_(*k*) ≠ 0	

Coupling	*y*_*i*_⇔*y*_*j*_	*y*_*i*_⇒*y*_*j*_ or *y*_*j*_⇒*y*_*i*_	*a*_*mi*_(*k*) ≠ 0, …, *a*_*jm*_(*k*) ≠ 0 or *a*_*mj*_(*k*) ≠0,…, *a*_*im*_(*k*) ≠ 0	Γ_*ij*_(*f*) ≠ 0
Spurious coupling		*y*_*m*_⇒*y*_*i*_ and *y*_*m*_⇒*y*_*j*_	*a*_*sm*_(*k*) ≠ 0, …, *a*_*is*_(*k*) ≠ 0 and *a*_*sm*_(*k*) ≠ 0,…, *a*_*js*_(*k*) ≠ 0	
